# Observational Study of Pulse Transit Time in Children With Sleep Disordered Breathing

**DOI:** 10.3389/fneur.2020.00316

**Published:** 2020-05-08

**Authors:** Michael P. Yanney, Andrew P. Prayle, Nicola J. Rowbotham, Miguel Kurc, Sean Tilbrook, Nabeel Ali

**Affiliations:** ^1^Sherwood Forest Hospitals Foundation Trust, Mansfield, United Kingdom; ^2^Division of Child Health, Obstetrics and Gynaecology, Queens Medical Centre, Nottingham University Hospitals, Nottingham, United Kingdom; ^3^NIHR Nottingham Biomedical Research Centre, Queens Medical Centre, Nottingham University Hospitals, Nottingham, United Kingdom

**Keywords:** pulse transit time, sleep disordered breathing, upper airway resistance syndrome, oximetry, sensitivity and specificity, children, video, sound

## Abstract

**Background:** Pulse transit time (PTT) is a non-invasive measure of arousals and respiratory effort for which we aim to identify threshold values that detect sleep disordered breathing (SDB) in children. We also compare the sensitivity and specificity of oximetry with the findings of a multi-channel study.

**Methods:** We performed a cross-sectional observational study of 521 children with SDB admitted for multi-channel sleep studies (pulse oximetry, ECG, video, sound, movement, PTT) in a secondary care centre. PTT data was available in 368 children. Studies were categorised as normal; primary snoring; upper airway resistance syndrome (UARS); obstructive sleep apnoea (OSA), and “abnormal other.” Receiver operator characteristic curves were constructed for different PTT (Respiratory swing; Arousal index) thresholds using a random sample of 50% of children studied (training set); calculated thresholds of interest were validated against the other 50% (test set). Study findings were compared with oximetry categories (normal, inconclusive, abnormal) using data (mean and minimum oxygen saturations; oxygen desaturations > 4%) obtained during the study.

**Results:** Respiratory swing of 17.92 ms identified SDB (OSA/UARS) with sensitivity: 0.80 (C.I. 0.62–0.90) and specificity 0.79 (C.I. 0.49–0.87). PTT arousal index of 16.06/ hour identified SDB (OSA/UARS) with sensitivity: 0.85 (95% C.I. 0.67–0.92) and specificity 0.37 (95% C.I. 0.17–0.48). Oximetry identified SDB (OSA) with sensitivity: 0.38 (C.I. 0.31–0.46) and specificity 0.98 (C.I. 0.97–1.00).

**Conclusions:** PTT is more sensitive but less specific than oximetry at detecting SDB in children. The additional use of video and sound enabled detection of SDB in twice as many children as oximetry alone.

## Introduction

Obstructive sleep disordered breathing (SDB) is a syndrome of upper airway dysfunction characterized by snoring and/or increased respiratory effort during sleep (1–3). It includes a spectrum of disorders: primary snoring (PS); upper airway resistance syndrome (UARS), obstructive hypoventilation, and obstructive sleep apnoea (OSA) (2, 3). There is strong evidence of adverse neurocognitive, behavioral and cardiovascular outcomes in children with SDB, underlining the importance of diagnosis and management (4–7). The mechanisms behind these adverse outcomes are also being elucidated (8).

History and examination are poor at discriminating between children with OSA and PS and unreliable for determining which children require treatment (9, 10). Polysomnography (PSG) is the gold standard test for diagnosis but availability is limited (2, 11). Polygraphy is a less invasive alternative to PSG but also remains the domain of tertiary centers. Oximetry is widely used in both secondary and tertiary care centers and has been shown to have good specificity for detecting OSA in children but is much less sensitive. A study by Brouillette et al. suggests the sensitivity of oximetry may be about 50% when compared to PSG, but more recent studies have shown a much improved sensitivity using newer artificial intelligence techniques (12–14). Brouillette et al. defined a desaturation as a decrease in SaO_2_ by 4% or more from baseline and a positive oximetry study as having three or more desaturation clusters with at least three desaturations < 90%. A cluster of desaturations was defined as five or more in a 10–30 min period; periods of artifact and wakefulness (based on heart rate variability criteria) were excluded from analysis. There is currently much interest in less invasive sleep study modalities or indices that can detect SDB with accuracy. A recent trend for some UK Clinical Care Commissioning Groups to refuse to fund adenotonsillectomies for SDB, unless the diagnosis has been confirmed with a sleep study, has also increased the importance of diagnostic accuracy (15).

We report the findings of using a limited multi-channel sleep system incorporating electrocardiogram (ECG), oximetry, video, sound, and pulse transit time (PTT) in a secondary care center. The VISI-sleep system was compared directly with PSG in a validation study by van Someren et al. in which 10 children aged 0.2–6.4 years were evaluated with both the Visilab system (oximetry, sound, video, and movement) and a conventional polysomnographic system (Oxcams), incorporating pulse oximetry, ECG, nasal airflow (thermistors), chest and abdominal movement (impedance), and video (16). There were just two discrepancies in the final diagnosis between the two systems. One child deemed to have a normal study with the Visilab system had mild obstruction identified with PSG. Another child deemed to have obstruction with the Visilab system was shown to have mixed apnoea with PSG. The authors also demonstrated good interobserver reliability in 17 sleep studies evaluated independently by two clinicians (*k* = 0.52). They concluded that the Visilab video system is easy to use and robust providing its limitations are understood.

The utility of PTT for detecting subcortical arousals in children with SDB is known, but values considered significant have not been established (17). We have evaluated two PTT indices (PTT arousals index; PTT respiratory swing) in children with suspected SDB to ascertain values that might be predictive of OSA. As a secondary objective, we have also assessed the sensitivity and specificity with which oximetry detects SDB compared with a multi-channel study. Our aim is to determine the value of the use of video and sound in addition to oximetry.

## Methods

**Figure 1:**
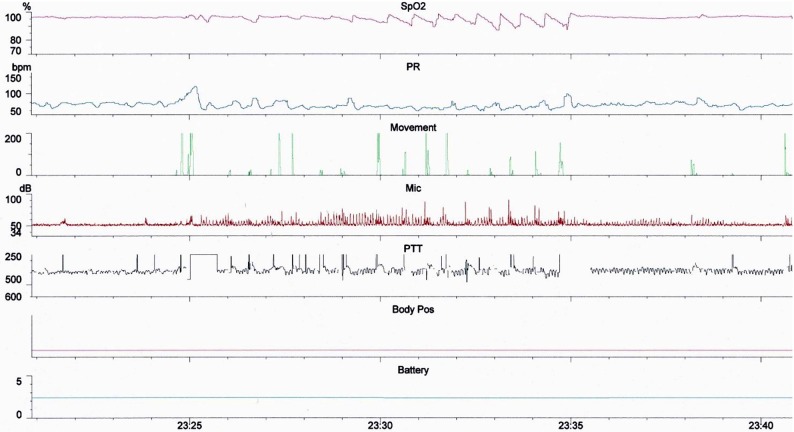
Example of a 20 min epoch of the multi-channel study montage.

We performed a cross sectional observational study of 581 children (<18 years of age) referred consecutively to a UK secondary care center for multi-channel sleep studies between February 2016 and November 2018. Children were referred predominantly for symptoms of SDB by otolaryngologists, pediatricians or general practitioners. Sleep studies were performed with two Stowood Scientific Instruments VISI-3 sleep systems incorporating ECG, video, sound, movement, pulse transit time and oximetry data (Figure 1). The VISI-3 sleep system utilizes Masimo technology for obtaining oximetry data with 2–4 s averaging times. Oximetry was measured with probes attached to either a finger or toe. Video was recorded with a Sony EVI-D90P infra-red camera. Children with symptoms of SDB who were unable or unwilling to undergo inpatient multi-channel studies had home oximetry studies instead. The data of these children were not included in the study.

**Figure 2:**
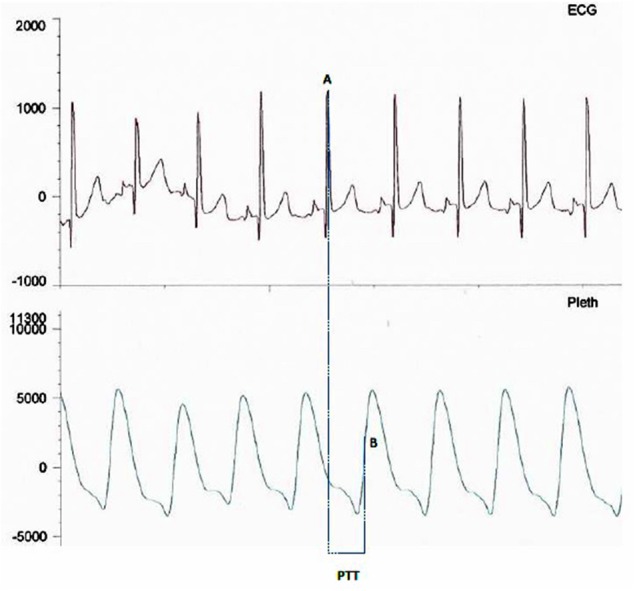
Image showing PTT measurement between mid-point of the R wave **(A)** on the ECG trace and mid-point of the up slope **(B)** on the plethysmography curve.

PTT is measured from the mid-point of the R wave of the QRS sequence obtained by electrocardiography (ECG) to a pulse waveform value obtained from an oximeter and measured at 50% of the maximum point of the plethysmography curve (Figure 2). A rise in mean arterial pressure (MAP) causes the pulse wave to travel faster and PTT to shorten; conversely a lower MAP causes PTT to lengthen. PTT is therefore an estimate of the time taken for the pulse pressure wave to travel from the aortic valve to the periphery and is inversely correlated to blood pressure (BP) changes (18). PTT can identify changes in inspiratory effort as a result of BP fluctuations induced by negative pleural pressure swings.

PTT is measured in milliseconds and is calculated with VISI-3 software; PTT2 was calculated by applying a 17 point averaging (~3.5 s) to the recorded raw channel PTT data. A PTT arousal was calculated as a drop in PTT2 >= 15 ms within 5–45 s as long as the PTT2 value was in the valid range of 150–500 ms. The PTT arousal index (PTT-AI) was calculated as the number of PTT arousals/ hour over the duration of the study. The average respiratory swing was analyzed using a derived PTT channel which had been interpolated for 1 s and then a three sample moving window average applied. The respiratory swing is the average size of respiratory rise from an inspiratory trough to expiratory peak and is measured in milliseconds.

### Exclusions

**Figure 3:**
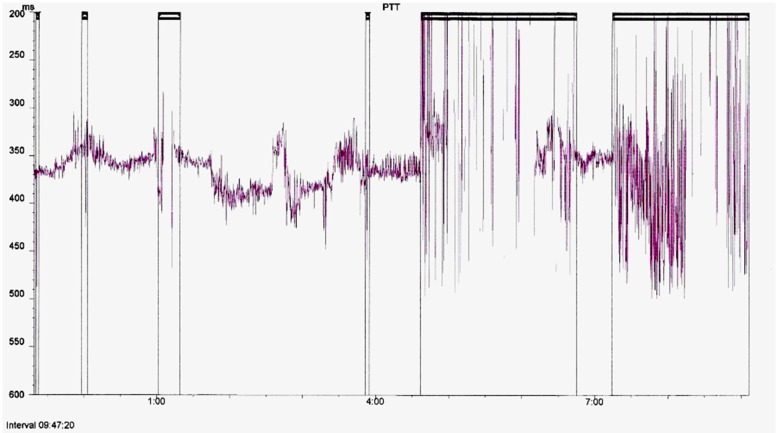
Example of a full study PTT montage with artifact highlighted for exclusion from analysis.

The PTT and oximetry traces were assessed for artifact by a sleep physiologist prior to reporting. Oximetry artifact associated with movement or low perfusion was excluded; PTT artifact resulting from either plethysmography or ECG signal dropout was also excluded. PTT artifact was identified as rapid spikes in excess of 50 ms; typically >100 ms (Figure 3). Children with <4 h artifact free oximetry data were excluded from the study and children with <3 h artifact free PTT data were excluded from the PTT analysis. We also excluded children categorized as “abnormal other” whose sleep study findings were due to causes unrelated to OSA, such as central apnoeas, chronic lung disease, a lower respiratory tract infection, or an exacerbation of asthma.

Sleep study categories were determined by a clinician (MY) using oximetry, video and sound criteria listed below. The oximetry, heart rate, sound and movement traces were used to identify sections of video that needed closer inspection. The video was assessed for evidence of obstructive episodes (defined below). Following assessment of the oximetry data, sleep study montage and video, the reporting clinician assigned one of the following five categories: normal; primary snoring; upper airway resistance syndrome; obstructive sleep apnoea or abnormal other. The reporting clinician remained blind to the PTT values until after the sleep study categories had been determined. Oximetry categories (Table 1) are based on those used by Sheffield Children's Hospital sleep service for perioperative risk stratification of children being considered for adenotonsillectomy. These categories are currently used in our hospital to guide management in children who have home oximetry and are the basis for determining which children might benefit from surgery and whether they are suitable for surgery in a secondary care center (12, 19–22).

**Table 1:** Oximetry risk criteria for OSA.

	**Normal**	**Inconclusive**			**Abnormal, low risk**	**Abnormal, high risk**
Baseline	>94% and		94% or		<94% or	<94% or
Desaturation Index (>4% dip from baseline)	<4/h and	>4/h and	or	<4/h and	>4/h and	>4/h and
Minimum saturation	>90%	>90%		80–90%	80–90%	<80%

#### Sleep Study Category Definitions

▪ **Normal**: No snoring or obstructed breathing evident on video and normal or inconclusive oximetry (Table 1)
▪ **Primary snoring**: Snoring but <3 witnessed obstructive episodes on video and normal or inconclusive oximetry
▪ **Upper airway resistance syndrome**: Video and sound evidence of 3 or more discrete periods of obstructed breathing, associated arousals and normal or inconclusive oximetry
▪ **Obstructive sleep apnoea**: Video and sound evidence of obstructed breathing, associated arousals and abnormal oximetry
▪ **Abnormal other**: Abnormal oximetry findings without any video evidence of obstruction.

#### Other Definitions

▪ **Obstructive episodes** were identified on video as periods when there was a pause in snoring but continued respiratory effort, followed by an airway opening noise and an arousal.
▪ **An arousal** was identified if there was movement associated with an obstructive episode and a corresponding pulse rate rise.

Weight was measured with SECA (Hamburg, Germany) electronic chair scales or SECA baby scales. Height was measured with a SECA wall mounted stadiometer or a Dunmow Rollameter (Harlow Healthcare, UK).

The sleep study categories, oximetry indices, pulse transit time indices, growth measures, age, sex and referring clinician were recorded prospectively in a database maintained for audit and service evaluation purposes. The following oximetry indices were recorded: mean saturation, minimum saturation, dip index defined as >4% drop in baseline saturation/ hour and lasting for > 5 s but <180 s, mean heart rate and standard deviation, oximetry categories (normal; inconclusive; abnormal low risk; abnormal high risk) and duration of artifact free oximetry data. The PTT respiratory swing, PTT arousal index (number of PTT arousals/ hour) and duration of artifact free PTT data were also recorded.

### Data Analysis

Receiver Operator Characteristic Curves (ROC) were calculated for PTT respiratory swing and PTT arousal index, using a random sample of 50% of the data (the training set). Cut offs were identified in the training set which had 90% sensitivity, 90% specificity, and a third cut off which maximized the arithmetic sum of sensitivity and specificity. To evaluate the performance of these thresholds in a second sample, they were validated against the other 50% (the test set), calculating the sensitivity and specificity for these thresholds. Data comparing sleep study outcomes based on oximetry findings or multi-channel study categories are presented in Table 2. Children classified as “Abnormal other” following multi-channel studies are not included in the table. We have calculated the sensitivity and specificity with which oximetry identified SDB compared to the multi-channel study. Data were analyzed with R (version 3.4) using the packages dplyr, beeswarm, and pROC (23–25). Confidence intervals were calculated with the inbuilt command in the pROC package using the bootstrap method to calculate the sensitivity and specificity. For the arithmetic sum, we added together the sensitivity and the specificity for each threshold of PTT-AI or PTT respiratory swing. We then used the maximum one as the best threshold; chosen to minimize mis-diagnosis (26). Z scores for weight and BMI were calculated using WHO growth standard and the package hgdb (27).

**Table 2:** Sleep study categories compared with oximetry findings.

**Oximetry categories**	**Number of children**	**Video findings**	
		**Normal or primary snoring**	**UARS or OSA**
Normal	196	153	43
Inconclusive	249	180	69
Abnormal	76	6	70

A retrospective evaluation of 176 children with SDB referred for sleep studies informed our PTT sample size estimate although a formal calculation was not done (28). We analyzed all available data for the 32 month period from February 2016 to November 2018 resulting in a final sample size of 368 children for the PTT analysis (190 children in the training dataset).

### Ethical Considerations

The Research and Development department at Sherwood Forest Hospitals Foundation Trust was approached before commencing data collection. They confirmed that ethical approval was not required for the analysis of anonymised sleep study data which involved no change to usual care (29).

## Results

**Figure 4:**
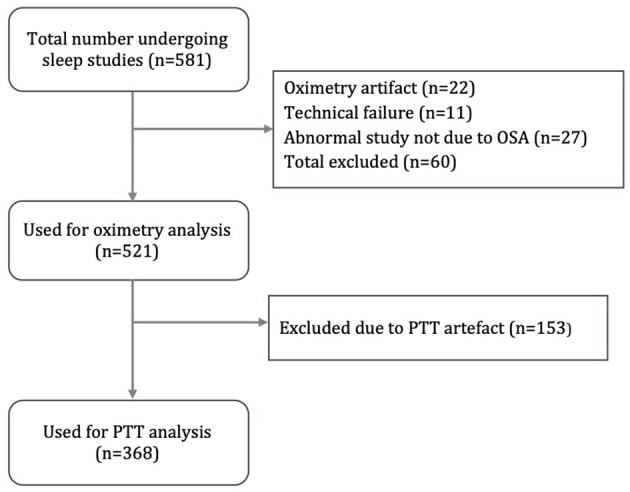
Flow diagram of study participants.

Figure 4 is a flow diagram of children assessed for the study. Oximetry and video data were available for 521 children (300 male; 221 female) and used for the oximetry sensitivity analysis. The data of 368 children (208 male; 160 female) were used for PTT analysis. The mean duration of oximetry data was 8.8 h (4.1–12) and the mean duration of PTT data was 5.1 h (3.0–10.5).

Table 2 shows the oximetry categories compared with the multi-channel study diagnoses. Use of a multi-channel study resulted in the detection of OSA or UARS in 182 children. Oximetry was abnormal in 76 children, 70 of whom had OSA. Oximetry was inconclusive or normal in 445 children, 112 of whom were diagnosed with UARS. Oximetry identified SDB (OSA) with a sensitivity of 0.38 (C.I. 0.31–0.46) and specificity 0.98 (C.I. 0.97–1.00); by definition, UARS cannot be identified with oximetry. Almost half (48%) of oximetry measurements (*n* = 249) were categorized as inconclusive.

**Figure 5:**
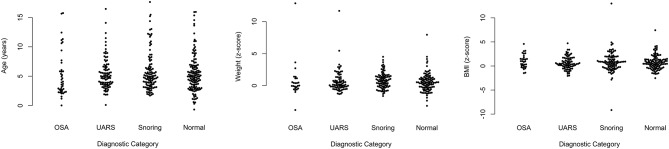
Scatter plots of age, weight z-scores and BMI z-scores according to diagnosis.

The age range of children included in the PTT analysis was as follows: five children were < 1 year; 188 were aged 1–4 years; 127 were 5–8 years; 33 were 9–12 years; and 15 were 13–17 years. The mean age of children with OSA was 5.22 years; standard deviation (SD) 3.73. For those with UARS, mean age was 5.45 (SD 2.69); for children with primary snoring, mean age was 5.8 (SD 3.3); and for those with a normal study, mean age was 5.4 (SD 3.2). Figure 5 shows scatter plots of age, weight z-scores, and body mass index z-scores demonstrating no significant differences in the baseline characteristics for any of the diagnostic categories.

**Figure 6:**
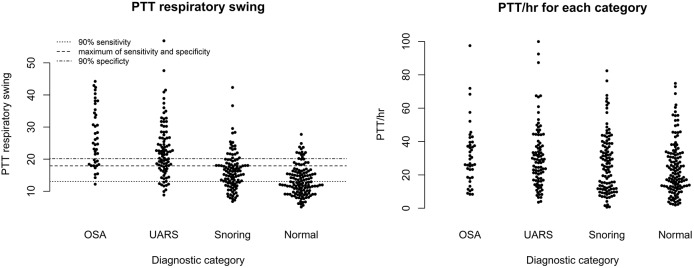
Scatter plots of PTT respiratory swing and PTT arousal index.

**Figure 7:**
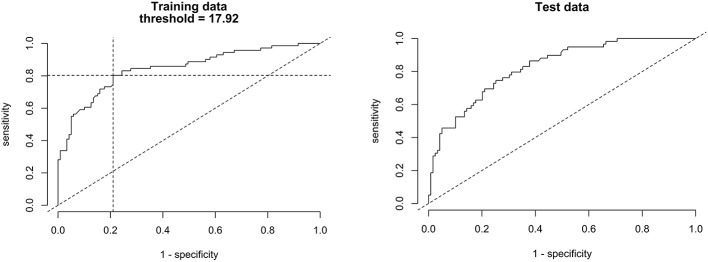
ROC curves of PTT respiratory swing (training and test data) compared with a multi-channel study used to detect OSA or UARS.

Figure 6 shows scatter plots of the PTT respiratory swing and PTT arousal index according to diagnostic categories showing higher respiratory swing values in the groups with UARS and OSA compared to those with a normal study or primary snoring. Figure 7 shows ROC curves of PTT respiratory swing (training and test datasets) and shows that a value of 17.92 ms maximized the sensitivity and specificity of detecting UARS or OSA, with a sensitivity of 0.80 (95% C.I. 0.62–0.90) and specificity of 0.79 (95% C.I. 0.49–0.87). Other thresholds of sensitivity and specificity are shown in Tables 3, 4. We replicated the ROC curves with the testing set and found a PTT respiratory swing of 17.91 ms detects UARS or OSA with a sensitivity of 0.75 and specificity of 0.72.

**Table 3:** 95% confidence intervals for PTT respiratory swing sensitivity at three thresholds (training data).

	**2.5%**	**50%**	**97.5%**
17.92 ms	0.62	0.77	0.90
13.07 ms	0.82	0.90	0.97
20.14 ms	0.46	0.61	0.76

**Table 4:** 95% confidence intervals for PTT respiratory swing specificity at three thresholds (training data).

	**2.5%**	**50%**	**97.5%**
17.92 ms	0.49	0.77	0.87
13.07 ms	0.30	0.46	0.79
20.14 ms	0.81	0.91	0.97

**Figure 8:**
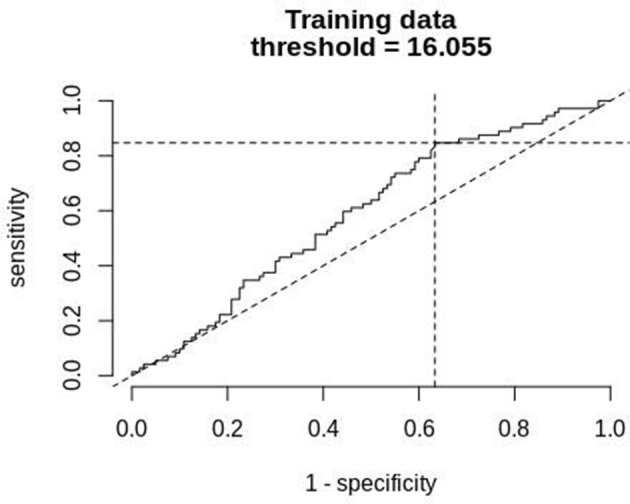
ROC curve of PTT arousal index (training data) compared with a multi-channel study used to detect OSA or UARS.

Figure 8 shows a ROC curve of the PTT arousal index demonstrating poor specificity for detecting UARS or OSA. A value of 16.06/ hour identified children with UARS or OSA with a sensitivity of 0.85 (95% C.I. 0.67–0.92) and specificity of 0.37 (95% C.I. 0.17–0.48). Other training data PTT thresholds with sensitivity, specificity and confidence intervals are shown in Tables 5, 6. Table 7 shows the number of children with PTT respiratory swing values above and below the 17.92 ms threshold within the combined diagnostic categories (UARS/ OSA and snoring/ normal) in both the training and test datasets.

**Table 5:** 95% confidence intervals for PTT arousal index sensitivity at the three thresholds of sensitivity (training data).

	**2.5%**	**50%**	**97.5%**
16.05/h	0.67	0.82	0.92
11.32/h	0.82	0.90	0.97
49.09/h	0.03	0.10	0.22

**Table 6:** 95% confidence intervals for PTT arousal index specificity at the three thresholds (training data).

	**2.5%**	**50%**	**97.5%**
16.05/h	0.17	0.34	0.48
11.32/h	0.09	0.22	0.42
49.09/h	0.82	0.90	0.98

**Table 7:** Number of children above and below PTT respiratory swing threshold in both training and test dataset.

	**Training data**			**Test data**			**Overall total**
	**Above PTT swing threshold**	**Below PTT swing threshold**	**Total**	**Above PTT swing threshold**	**Below PTT swing threshold**	**Total**	
UARS or OSA	57	14	71	44	15	59	130
Normal or snoring	24	95	119	33	86	119	238
Total	81	109	190	77	101	178	368

There were 177 children with normal or inconclusive oximetry who had PTT data available for analysis; 121 had a normal study or primary snoring using multi-channel criteria and 96 of 121 had a PTT respiratory swing below 17.92 ms. In the subgroup of children with normal or inconclusive oximetry, there were 56 with a diagnosis of UARS, 43 of whom had PTT respiratory swing values above 17.92 ms. Thus, when oximetry was inconclusive, PTT respiratory swing gave useful information on the presence or absence of UARS (χ^2^ test *p* < 0.001).

## Discussion

This study is to our knowledge, the largest to evaluate PTT in children with SDB. A systematic review by Smith et al. evaluated 21 studies of the use of PTT in children. Most studies used PTT to screen for OSA whilst a few evaluated the use of PTT as a surrogate measure to track changes in blood pressure. The PTT arousal index (PTT-AI) is the most commonly studied PTT parameter and is a measure of the number of defined changes in PTT/ hour (17).

Pitson et al. have used “PTT swings” (Respiratory swing) in their evaluation of eight patients aged 13–68 years with OSA who were being started on nasal continual positive airway pressure (30). Their aim was to ascertain whether respiratory oscillations in PTT could provide a useful measure of changes in respiratory effort in patients with sleep related breathing disorders. Patients were monitored with esophageal manometry, electroencephalograms (EEG), infra-red video, sound, oximetry and pulse rate. The authors found excellent correlation between the size of the swings in esophageal pressure and the size of the PTT swings (mean r=0.94), confirming its usefulness as a non-invasive measure of respiratory effort. Mehendale et al. also measured “PTT inspiratory effort” (Respiratory swing) in a study of 44 children with velopharyngeal incompetence, before and after a Sommerlad palate repair or a Hynes pharyngoplasty (31). Children were assessed for OSA with multi-channel cardiorespiratory studies and PTT inspiratory effort was used to assess changes in respiratory effort pre and post-operatively. The authors found a significant increase in PTT inspiratory effort (*p* = 0.04) in children undergoing a Hynes pharyngoplasty, suggesting increased upper airway resistance and respiratory effort. A significant increase in the obstructive sleep apnea/hypopnea grading was also noted post-operatively (*p* = 0.002). The authors suggest that PTT inspiratory effort may have particular relevance in the identification of children with mild OSA and increased upper airway resistance resulting in increased inspiratory effort. Our findings are consistent with those of Mehendale et al.

A study by Katz et al. found PTT arousal index to be a more sensitive marker of cortical arousals than EEG and esophageal manometry (18). Griffon et al. also found PTT useful for discriminating between obstructive and central apnoeas but noted that about a third of apnoeas could not be scored due to artifact (32). We encountered similar problems with PTT artifact, leading to the exclusion of 26% of data. PTT is particularly prone to artifact due to the fact that it is computed from two physiological signals. Artifact occurs as a result of interference with the photoplethysmographic signal at the toe or finger or from loss of ECG signal due to disruption of chest wall leads with movement. Rapid eye movement (REM) sleep is particularly prone to PTT artifact due to significant variations in respiratory drive and rapid fluctuations in pulse rate and blood pressure affecting stability of the PTT signal. Obstructive events are most likely to occur during REM sleep and so a loss of stability in the PTT signal at this stage of the sleep cycle amplifies the impact of artifact.

Studies by Brietzke et al. and Bradley et al. have assessed the utility of PTT-AI to detect SDB in children using PSG as the gold standard (33, 34). Brietzke et al. studied 59 unselected symptomatic children routinely scheduled for PSG, 11 of whom had previously had an adenotonsillectomy and 15 who had a craniofacial syndrome (trisomy 21 and Apert syndrome) (34). The authors found that a PTT-AI cut off of 7.4 events/ hour identified SDB, defined as an Apnoea Hypopnea Index (AHI) > 3, with 93% sensitivity and 91% specificity. A PTT-AI cut off of 5.4 events/ hour identified SDB; defined as AHI > 1, with 81% sensitivity, and 76% specificity. The authors concluded that PTT-AI had excellent utility for detecting moderate or severe SDB but was not significantly better than oximetry at detecting mild OSA. A similar study by Bradley et al. of 51 children aged 5–17 years with suspected SDB, found that a PTT-AI cut off of 11.36 events/ hour identified OSA (defined as AHI >3) with 94% sensitivity and 62% specificity. The same PTT-AI cut off identified OSA (defined as AHI >1) with a sensitivity of 66% and specificity of 67%. The authors showed that PTT-AI has validity for detecting moderate to severe OSA (AHI>3) but not mild OSA (33). In this study we found a poor association between PTT-AI and OSA or UARS, probably because about 60% of our cohort could be categorized as mild OSA (defined as UARS in this study). We observed that restless sleep may confound the ability of PTT-AI to detect SDB in our cohort. We did, however, find an association between PTT respiratory swing and OSA/UARS. There is emerging evidence that children with mild OSA (or UARS) have an increased risk of neurocognitive impairment, due to the effects of sleep fragmentation associated with frequent subcortical arousals in the absence of intermittent hypoxia (8, 35, 36). There is currently much interest in the identification of sleep study indices or biomarkers that can accurately identify children who would benefit from treatment (4). Further work is needed to ascertain whether PTT respiratory swing identifies children with mild SDB who are at risk of adverse neurocognitive outcome and who would benefit from adenotonsillectomy or treatment with nasal corticosteroids +/– leukotriene antagonists (37–40).

A potential limitation of this study is our use of SDB definitions (OSA, UARS, PS) based on multi-channel study criteria (oximetry, movement, video and sound) rather than PSG. The sleep system used (Stowood Scientific Instruments VISI−3 sleep system) records standard signals utilizing established and validated methods. For oximetry data, the sleep system utilizes Masimo technology which is validated in children and is favored due to its effectiveness at detecting and excluding motion artifact and because it has the option of a short averaging time (41, 42). PTT data was obtained with VISI software, using the same (or an upgraded version of) equipment as that used in several studies of PTT in children aged 1–18 years using PSG and/ or esophageal manometry as the comparator (16, 18, 30, 31, 33, 34, 43). A validation study by van Someren et al. of the Visilab system in a pediatric clinical setting, found the results comparable to those from PSG and with good interobserver reliability (16).

A study by Brouillette et al. showed that oximetry criteria for the diagnosis of OSA correlated with PSG findings with 98% specificity but 43% sensitivity (12). We found that abnormal oximetry correlated with multi-channel study findings of OSA with 98% specificity and 38% sensitivity; similar to the findings of Brouillette et al. using PSG as the comparator. The diagnosis of OSA in this study was based on both oximetry and video criteria making it likely that the findings are robust.

The oximetry criteria we have used to diagnose OSA have been validated in several studies (12, 19–22). We are therefore confident that children categorized as OSA in this study would correlate well with PSG given the known positive predictive value of oximetry and the additional video confirmation of the findings (12). We would also consider our definition of a normal study to be valid based on the absence of snoring, no obstructive events evident on video recording and no abnormal oximetry criteria. We acknowledge that our categories of UARS and PS need further validation. The strict definition of UARS requires esophageal manometry, an invasive technique which is not routinely used in the evaluation of children with SDB (18). The European Respiratory Society (ERS) task force statement defines UARS as follows: snoring, increased work of breathing, frequent arousals, but no recognizable obstructive events or gas exchange abnormalities (2). Our use of the term UARS is in line with these criteria apart from the identification of obstructive events on video. Based on the video evidence of obstructive episodes in children in this category, we presume that PSG will demonstrate some airflow obstruction, although it is uncertain if all witnessed obstructive events would meet the scoring criteria for apnoeas or hypopnoeas. We think it is likely that a number of children categorized as UARS would meet PSG criteria for mild OSA (Apnoea-Hypopnea Index 1–5). Our definition of UARS uses the same audio/video criteria as that used to confirm OSA; the main difference between the groups being the inconclusive or normal oximetry in those with UARS and abnormal oximetry in those with OSA.

The category of primary snoring is based on snoring being evident on sound recording with no (or minimal) evidence of obstructive events on video (2 or fewer events) and no abnormal oximetry criteria. The video criteria used to identify obstructive episodes have previously been described (44, 45). It is probable that some children in our categories of PS or UARS would be reclassified with PSG. We would argue however, that within the limitation of studies incorporating oximetry, video and sound, there is a need for distinction between the range of categories we have identified to aid clinical management decisions and particularly to identify children with OSA who are missed by the use of oximetry alone. It is possible that different labels could be used for the UARS category such as “probable OSA” or “video diagnosed OSA.” The use of the term UARS in clinical practice makes it possible to convey the essence of what clinicians need to know - that children with this diagnosis need more careful evaluation because some may benefit from surgery, whilst others could be managed with medical therapies (nasal corticosteroids +/– leukotriene antagonists) or watchful waiting. We note the striking similarities in PTT respiratory swing values in the categories of OSA and UARS (Figure 6) and the similar PTT respiratory swing values for children with PS or a normal study. We believe this observation provides some validity for our view that our categories of UARS and PS identify different entities within the SDB spectrum.

We sought to maintain consistency in our diagnosis of UARS by ensuring we identified three discrete clusters of obstructed breathing during the study period. This is based on oximetry criteria reported by Brouillette et al. recommending three or more clusters of desaturation episodes are identified for the diagnosis of OSA (12). We consider the UARS definition we have used to be pragmatic and potentially useful in health care settings with no access to PSG. Most children with SDB in the UK do not have access to PSG to confirm a diagnosis or to aid treatment decisions (46). Oximetry is the most widely used modality to identify SDB in children seen in UK secondary care centers but may only detect about 50% of affected children (12). We therefore surmise that many children with SDB and at risk of adverse neurocognitive outcomes remain undiagnosed. Furthermore, due to current trends in health care funding, these children may be unable to access necessary treatment (15).

We have demonstrated the feasibility of using a limited multi-channel sleep system in a secondary care center and have shown how the use of video and sound in addition to oximetry, increased the detection of SDB 2-fold compared to oximetry alone. It is our local experience that the findings from a multi-channel system are useful to Ear, Nose, and Throat (ENT) surgeons who are able to offer surgery and can expect to receive funding for children found to have UARS. Many are also managed with watchful waiting. However, if ENT surgeons only have a normal or inconclusive oximetry result on which to base management decisions, they are less likely to offer adenotonsillectomy in children with symptoms of SDB. This may be a direct result of the recent trend for Clinical Care Commissioning Groups to refuse funding for adenotonsillectomies in children with symptoms of SDB, who have no objective confirmation of OSA. This study also identifies the potential for PTT used in combination with oximetry to significantly improve the detection of SDB. This finding may have wide applicability but may be of particular interest to secondary care centers with no access to PSG.

We avoided bias by ensuring the PTT data analysis was done by a sleep physiologist and only reviewed by the reporting clinician after the sleep study categories had been determined. The reporting clinician was therefore blind to the PTT values whilst determining sleep study categories. The use of predetermined oximetry and video criteria for sleep study categories also minimized bias.

We envisage that PTT could be a useful addition to home multi-channel studies which are increasingly being evaluated for the benefits they afford over inpatient polygraphy or PSG. When given a choice, parents prefer home multi-channel studies to inpatient polygraphy or PSG for their convenience and because they facilitate better quality sleep; they also have potential cost benefits by avoiding an overnight hospital stay (47, 48). However, a significant challenge for home multi-channel studies is the problem of artifact. Our use of PTT monitoring in a hospital setting, where dislodged leads were resited during the night, still resulted in considerable artifact. This may be a limiting factor for PTT use in home multi-channel studies.

It is likely that oximetry obtained as part of inpatient multi-channel studies has reduced sensitivity for detecting SDB compared with home oximetry. Home studies potentially allow better quality sleep with more time in REM sleep, when SDB is mostly likely to occur.

The use of parental home video recordings has been shown to be a reliable screening tool for OSA and has good correlation with PSG findings (44). Parental home videos are currently being evaluated along with questionnaires to identify children who might be offered surgery for OSA without formal sleep studies.

This study demonstrates the potential for less invasive modalities to achieve a significant degree of accuracy in the detection of SDB. It will be important to validate the PTT findings with PSG or other treatment outcomes (i.e., neurocognitive).

## Conclusions

We conclude that PTT respiratory swing can help identify SDB in children and is more sensitive but less specific than oximetry. We also found that the additional use of video and sound increased the detection of SDB 2-fold compared with oximetry alone. PTT used in combination with oximetry (+/– video and sound) could significantly improve the detection of SDB in health care settings with limited or no access to PSG.

## Data Availability Statement

The datasets generated for this study are available on request to the corresponding author.

## Ethics Statement

Ethical review and approval was not required for the study on human participants in accordance with the local legislation and institutional requirements. Written informed consent from the participants' legal guardian/next of kin was not required to participate in this study in accordance with the national legislation and the institutional requirements.

## Author Contributions

MY was responsible for the original concept and design of the study, data collection, some data analysis and produced the first draft of the manuscript. AP and NR performed most of the data analysis. MK and ST were involved in data collection. NA contributed to the original concept and design of the study. All authors contributed to the manuscript and approved the final version.

## Conflict of Interest

NR has given talks at events sponsored by TEVA. The remaining authors declare that the research was conducted in the absence of any commercial or financial relationships that could be construed as a potential conflict of interest.

## Plain Language Summary

Pulse transit time (PTT) is a measurement calculated from the ECG and oximetry traces obtained during a sleep study, which has previously been shown to have the potential to help diagnose sleep disordered breathing (SDB). In this study we have identified threshold PTT values associated with SDB in children. Our findings suggest PTT is more sensitive but less specific than oximetry at detecting SDB. We also found that by combining video, sound and oximetry, we detected SDB in twice as many children as oximetry alone.
